# The HPV-TP53-MALAT1 Axis: Unravelling interactions in cervical cancer development

**DOI:** 10.1371/journal.pone.0291725

**Published:** 2023-10-09

**Authors:** Saba Iordanishvili, Tornike Metreveli, Elene Lipartia, Konstantine Gachechiladze, Irakli Khuntsaria, Tamar Qobulashvili, Mariam Jorbenadze, Tamaz Revazishvili, Ekaterina Kldiashvili, Andreas Martin Kaufmann

**Affiliations:** 1 Petre Shotadze Tbilisi Medical Academy, Tbilisi, Georgia; 2 Charite Universitatsmedizin Berlin, Berlin, Germany; UFRN: Universidade Federal do Rio Grande do Norte, BRAZIL

## Abstract

**Introduction:**

Cervical cancer, primarily driven by Human Papillomavirus (HPV) infection, stands as a substantial global health challenge. The TP53 gene’s, Arg72Pro polymorphism has emerged as a noteworthy player in cervical cancer development, particularly among individuals harboring high-risk (HR) HPV types. Additionally, long non-coding RNAs (lncRNAs), exemplified by metastasis-associated lung adenocarcinoma transcript 1 (MALAT1), exert critical roles in cancer biology. This study delves into unravelling the intricate connections linking HPV infection, TP53 Arg72Pro polymorphism, and MALAT1 expression in the context of cervical cancer.

**Materials and methods:**

Within a cohort of cervical cancer patients, we discerned HPV infection statuses, executed genotyping for the TP53 Arg72Pro polymorphism, and quantified MALAT1 expression through quantitative reverse transcription-polymerase chain reaction (qRT-PCR). Statistical analyses meticulously probed relationships intertwining HPV infection, TP53 polymorphism, and MALAT1 expression.

**Findings:**

Our investigation revealed a striking prevalence of the TP53 Arg72Pro polymorphism among HPV-positive subjects, accompanied by a robust and statistically significant correlation linking MALAT1 overexpression (p<0.01) and HR-HPV positivity (p<0.03). Importantly, a subset of MALAT1 overexpression cases unveiled a concomitant TP53 Pro72Pro polymorphism. In contrast, HPV-negative invasive cervical carcinoma samples exhibited no discernible shifts in MALAT1 expression.

**Conclusion:**

The contours of our findings sketch a compelling landscape wherein HR-HPV infection, TP53 polymorphism, and MALAT1 expression intertwine significantly in cervical cancer. The voyage ahead entails delving deeper into molecular underpinnings to decipher MALAT1’s nuanced role and its dance with TP53 within HPV-associated cervical carcinogenesis. This expedition promises insights that may engender targeted therapeutic interventions and bespoke prognostic markers, tailored to the realm of HR-HPV-related cervical cancer.

## Introduction

Cervical cancer remains a formidable global health challenge [1], driven by the central role of Human Papillomavirus (HPV) infection in its etiology [2]. Despite advancements in vaccination, the escalating prevalence of HPV-associated cancers underscores the imperative for more comprehensive investigations [3–5]. Unravelling the intricate molecular mechanisms underpinning HPV-associated cervical cancer progression assumes paramount importance for devising targeted interventions and augmenting patient outcomes. The HPV life cycle entails the infection of basal epithelial stem cells, intricately synchronized with viral oncogene expression and cellular differentiation to orchestrate virion production. Perturbations, such as promoter methylation obstructing E2 repressor binding or functional inactivation arising from E2 gene deletion or mutation, culminate in elevated expression of viral oncoprotein E6 and E7. These oncoproteins, targeting pivotal tumor suppressors p53 and pRb, crucially steer cellular transformation and cancer progression [6]. The disruption of control mediated by these fundamental cellular regulators precipitates unbridled growth and malignant transformation. Notably, a key genetic variant within the TP53 gene, the Arg72Pro polymorphism, has garnered considerable attention in the context of cervical cancer. Insightful research has disclosed the Arg72 variant’s heightened affinity for binding to the high-risk HPV E6 protein, distinguishing it from the Pro72 variant [7]. This intriguing observation suggests that the TP53 Arg72 variant might exert a pivotal role in both the inception and advancement of cervical cancer, especially within the cohort affected by HPV infection. Nevertheless, the impact of this polymorphism on cervical cancer outcomes in HPV-negative individuals remains shrouded in ambiguity, thus warranting deeper scrutiny.

Moreover, the emerging landscape of long non-coding RNAs (lncRNAs) in cancer biology introduces further layers of complexity. Among these, metastasis-associated lung adenocarcinoma transcript 1 (MALAT1), an infrequently spliced lncRNA, has surfaced as a significant player within various gynecological malignancies [8]. Dysregulation of MALAT1 associates with heightened cell proliferation, metastasis, and alteration of target gene splicing [9]. The intricate interplay between MALAT1 and the tumor suppressor TP53 confounds the molecular panorama of cervical cancer progression.

In this study, our objective was to meticulously scrutinize the potential interplay among HPV infection, the TP53 Arg72Pro polymorphism, and the lncRNA MALAT1 expression.

## Materials and methods

A total of 200 women within the reproductive age range (18–65 years) were enrolled in this study between April 14, 2019, and December 14, 2019. Among these, 150 women constituted the target group testing positive for high-risk HPV types (16/18/31/33/35), while the remaining 50 women comprised the control group, testing negative for HPV. Cervical cancer screening involved liquid-based cytology using Hologic Inc.’s system (Rome, Italy) for both groups, with details of HPV status and cytology results previously published and presented in Table 1 [10].

**Table 1:** Data on the HPV status and liquid-based cytology results for cervical cancer screening from the study participants.

Liquid-based cytology for cervical cancer screening (The 2001 Bethesda system)	Subjects (n)	HPV status: positive for high-risk HPV types 16/18/31/33/35
NILM: Negative for intraepithelial lesion or malignancy	42	No
ASC-US: Atypical squamous cells of undetermined significance	8	No
	57	Yes
LSIL: Low-grade squamous intraepithelial lesion	36	Yes
HSIL: High-grade squamous intraepithelial lesion	32	Yes
ASC-H: Atypical squamous cells, cannot exclude high-grade squamous intraepithelial lesion	25	Yes

Given the inherent stability of human nucleic acids in liquid-based cytology media [11], TP53 gene status was assessed through polymerase chain reaction-restriction fragment length polymorphism (PCR-RFLP) analysis using DNA extracted from these samples. Similarly, the expression of the lncRNA MALAT1 was evaluated in the same samples. Nucleic acid extraction followed by manufacturer’s protocols using DNA and RNA kits from Qiagen (Hilden, Germany), and extracted samples were stored at -18°C.

For TP53 Arg72Pro polymorphism determination, PCR-RFLP and BstUI restriction endonuclease (Fermentas, Vilnius, Lithuania) were employed. Amplification of a targeted 296-base pair (bp) fragment utilized the following primers:

**Forward**: 5’–TTGCCGTCCCAAGCAATGGATGA– 3’
**Reverse**: 5’–TCTGGGAAGGGACAGAAGATGAC– 3’.

Each 50 μL PCR reaction contained 0.1 μg genomic DNA; 1 U Taq DNA polymerase; 10 pmol of each primer; 200 mM of each deoxynucleotide triphosphate (dNTP); 1.5 mM magnesium chloride (MaCl2), with primers and reagents procured from Norgen Biotek Inc., (Thorold, Canada). The PCR conditions comprised an initial 5 min denaturation at 95°C, 30 cycles of 30 sec at 95°C, 30 sec at 57°C, and 1 min at 72°C, followed by a final step at 72°C for 7 min. Subsequent BstUI digestion of the resulting 312-bp PCR products of TP53 exon 4 adhered to the manufacturer’s guidelines (Fermentas, Vilnius, Lithuania). After separation via 4% agarose gel electrophoresis with ethidium bromide, digestion products were visualized using an ultraviolet (UV) transilluminator.

Reverse transcription of lncRNA MALAT1 RNA involved the high-capacity RNA-to-cDNA kit (Applied Biosystems, Foster City, CA, USA) in a 20 μL reaction volume, with inclusion of a negative control in each experiment to verify absence of genomic DNA contamination. All cDNA samples were stored at −20°C until further molecular analysis. The MALAT1 primers were:

**Forward**: 5′-CCCCACAAGCAACTTCTCTG-3′,
**Reverse**: 5′-TCCAAGCTACTGGCTGCATC-3′.

Quantitative reverse transcription-polymerase chain reaction (qRT-PCR) measured MALAT1 expression in a 10 μL reaction containing 1 μL of cDNA, 2 μL of PCR Synthesis Buffer, 1 μL of MgCl2, 0.2 μL of dNTPs, 0.5 μL of BSA, 0.1 μL of Hot Start DNA polymerase, 0.3 μL of forward and reverse primers, and 1 μL of 1X LC Green^®^. The qRT-PCR protocol encompassed an initial cycle at 95°C for 2 min, followed by 45 cycles of amplification at 95°C for 10 sec, 60°C for 10 sec, and 72°C for 10 sec. The melting curve analysis introduced a rapid cooling cycle to 40°C for 30 sec, with real-time fluorescence acquisition at the elongation step (72°C). Subsequent melting curve analysis incorporated steps at 55°C for 20 sec, 95°C for 20 sec, ramping at 0.19°C/sec, with continuous acquisition mode and the final step at 40°C for 10 sec. The reference gene, beta-2-microglobulin (B2M) was included, utilizing validated qRT-PCR assays [12]. Standardization of MALAT1 expression values employed B2M as the reference gene. Relative quantification (RQ) involved the ΔΔCq method [12], with the fold change of MALAT1 expression relative to the reference gene calculated using the formula RQ = 2^(-ΔΔCq).

Statistical analysis employed SPSS v.28.0.1.1 software, with intra-group comparisons determining p-values; significance was established at p<0.05. Ethical clearance was secured from the Bioethics International Committee of the Petre Shotadze Tbilisi Medical Academy (identification code: 20042019/2, Tbilisi, Georgia). All procedures adhered to the Helsinki Declaration of 1975, revised in 2013, with participants receiving comprehensive study information and providing written informed consent prior to inclusion.

## Results

This study sought to elucidate potential connections among HR-HPV infection, the TP53 Arg72Pro polymorphism, and lncRNA MALAT1 expression. Through TP53 Arg72Pro polymorphism analysis, we identified three distinct variants:

1. Homozygous wild-type Arg/Arg variant, characterized by bands of 259 bp and 53 bp.
2. Homozygous Pro/Pro variant, evident as a single band of 312 bp.
3. Heterozygous variant, signified by the presence of all three bands (259 bp, 53 bp and 312 bp).

Among HPV-positive subjects, the TP53 gene codon 72 polymorphism was identified in 142 cases (94.7%). Within this group, 102 individuals (68.0%) were identified as Pro/Pro homozygotes, and 40 individuals (26.7%) exhibited Arg/Pro heterozygosity. Detailed information on HPV status, liquid-based cytology results, and TP53 Arg72Pro polymorphism can be found in Table 2.

**Table 2:** HR-HPV status, liquid-based cytology result and TP53 Arg72Pro polymorphism frequency from the study groups.

Liquid-based cytology for cervical cancer screening (The 2001 Bethesda system)	Subjects (n)	TP53 Arg72Pro Polymorphism			HPV status: positive for high-risk HPV types 16/18/31/33/35
		Arg72Arg	Arg72Pro	Pro72Pro	
NILM: Negative for intraepithelial lesion or malignancy	42	40	2	—	No
ASC-US: Atypical squamous cells of undetermined significance	8	—	8	—	No
	57	6	12	39	Yes
LSIL: Low-grade squamous intraepithelial lesion	36	—	8	28	Yes
HSIL: High-grade squamous intraepithelial lesion	32	—	3	29	Yes
ASC-H: Atypical squamous cells, cannot exclude high-grade squamous intraepithelial lesion	25	2	17	6	Yes
Total		48	50	102	

Among the HPV-positive samples, 66.7% (100 out of 150) exhibited MALAT1 overexpression. Notably, only 15% of these cases were associated with TP53 Arg72Pro polymorphism, implying that a significant majority, 85%, of MALAT1 overexpression instances are linked to TP53 Pro72Pro polymorphism (Table 3). Conversely, HPV-negative samples did not demonstrate significant alterations in MALAT1 expression.

**Table 3:** MALAT1 overexpression and TP53 Arg72Pro polymorphism from the HPV-positive group.

Liquid-based cytology for cervical cancer screening (The 2001 Bethesda system)	Subjects (n)	TP53 Arg72Pro Polymorphism			MALAT1 overexpression
		Arg72Arg	Arg72Pro	Pro72Pro	
ASC-US: Atypical squamous cells of undetermined significance	57	6	12	39	27
LSIL: Low-grade squamous intraepithelial lesion	36	—	8	28	35
HSIL: High-grade squamous intraepithelial lesion	32	—	3	29	32
ASC-H: Atypical squamous cells, cannot exclude high-grade squamous intraepithelial lesion	25	2	17	6	6
Total		8	40	102	100

## Discussion

Our study aimed to unravel the intricate interplay between HPV infection, the TP53 Arg72Pro polymorphism, and the expression of the long non-coding RNA (lncRNA) MALAT1, particularly within the context of cervical dysplasia progression. MALAT1, a molecule of intense investigation across various cancers, including lung, endometrial, breast, and invasieve cervical cancer, initially surfaced as a potential as a prognostic marker for non-small cell lung cancer metastasis [13]. Its subsequent roles in diverse malignancies, marked by hyperproliferation and metastasis, have garnered attention [14–16]. Functionally, MALAT1’s involvement extends to cell cycle regulation, as exemplified by its interaction with unmethylated PC2 protein in HeLa cells, facilitating SUMOylation of E2F1, a critical cell cycle control transcription factor [17]. Moreover, insights from human diploid fibroblasts underscore MALAT1’s influence on G1 phase progression, hinting at its pivotal role in orchestrating cell cycle succession [18]. The precise mechanism by which MALAT1 modulates E2F1 activity, alongside potential interplays with p53-mediated cell cycle arrest, beckon further exploration [18]. Notably, recent time course experiments suggest a potential connection between MALAT1 and p53, with MALAT1 depletion potentially affecting p53 levels in response to double-stranded DNA damage, offering tantalizing clues about their intricate interplay [18]. Transcriptional regulation of MALAT1 complex narrative involves its interplay with the tumor suppressor protein p53. Insights reveal direct binding of p53 to specific binding motifs in MALAT1’s promoter region, leading to its transcriptional inhibition. Activated p53, often in response to cellular stress or DNA damage, contributes to downregulating MALAT1 expression [9].

Our findings underscore the TP53 Arg72Pro polymorphism’s prevalence among HPV-positive subjects, with 94.7% harboring this variant, comprising 68.0% Pro/Pro homozygotes and 26.7% Arg/Pro heterozygotes. Intriguingly, our analysis of MALAT1 overexpression and HPV status unveils 66.7% of HPV-positive samples exhibit heightened MALAT1 expression. Of note, a significant 85% of cases with MALAT1overexpression are associated with TP53 Pro72Pro polymorphism. In contrast, a smaller fraction (15%) of HPV-positive cases with MALAT1 overexpression exhibited TP53 Arg72Pro polymorphism. This pattern is concordant with previous observations and suggest a robust connection between HPV infection, TP53 polymorphism and MALAT1 overexpression, particularly in cervical tissues of experiencing escalating dysplasia. We posit that the TP53 Pro72Pro polymorphism potentially fosters p53 inactivation, unleashing uncontrolled MALAT1 transcription–a potential keystone culminating in transformation and cancerogenesis upon HR-HPV infection. However, the intricate molecular underpinnings driving MALAT1 dysregulation within HPV-related cervical carcinogenesis warrant nuanced exploration. Deciphering these mechanisms stand to illuminate pathogenic insights and unveil promising therapeutic avenues for both HPV-associated cervical cancer and its premalignant stages.

## Conclusion

Our study sheds light on the intricate relationship between HPV infection, the TP53 Arg72Pro polymorphism, and the heightened expression of the long non-coding RNA (lncRNA) MALAT1 in cervical tissues with associated dysplasia. Extensively explored in various cancers MALAT1’s propensity for fostering hyperproliferation and metastasisis noteworthy. Notably, our findings reveal a predominance of TP53 Pro72Pro polymorphism among HPV-positive subjects, correlated with increased MALAT1 expression. This hints at a plausible role of TP53 Pro72Pro polymorphism in unhindered MALAT1 transcription upon HPV infection, potentially fueling cervical carcinogenesis. While our study furnishes valuable insights into the intricate interplay between HPV infection, TP53 polymorphism, and MALAT1 overexpression, unravelling the molecular tapestry underlying these connections beckons further exploration. The enigmatic MALAT1 dysregulation within HPV-related cervical carcinogenesis merits in-depth investigation, offering a potential avenue for identifying therapeutic targets tailored to HPV-associated cervical cancer.

## Supporting information

S1 Checklist Human participants research checklist. (DOCX)

## References

[1] World Cancer Research Fund International. (n.d.). Worldwide Cancer Data. https://www.wcrf.org/cancer-trends/worldwide-cancer-data/

[2] Fontan C. T., James C. D., Prabhakar A. T., Bristol M. L., Otoa R., Wang X., et al. (2018). A Critical Role for p53 during the HPV16 Life Cycle. Microbiology Spectrum, 6(5), GPP3-0046-2018. doi: 10.1128/spectrum.00681-22 35608342 PMC9241828

[3] de Martel C., Plummer M., Vignat J., & Franceschi S. (2017). Worldwide burden of cancer attributable to HPV by site, country and HPV type. International Journal of Cancer, 141(4), 664–670. doi: 10.1002/ijc.30716 28369882 PMC5520228

[4] Marur S., D’Souza G., Westra W. H., & Forastiere A. A. (2010). HPV-associated head and neck cancer: A virus-related cancer epidemic. The Lancet Oncology, 11(8), 781–789. doi: 10.1016/S1470-2045(10)70017-6 20451455 PMC5242182

[5] Fakhry C., Lacchetti C., Rooper L. M., Jordan R. C., Rischin D., Sturgis E. M., et al. (2018). Human papillomavirus testing in head and neck carcinomas: ASCO clinical practice guideline endorsement of the College of American Pathologists Guideline. Journal of Clinical Oncology, 36(31), 3152–3161. doi: 10.1200/JCO.18.00684 30188786

[6] Idres Y. M., McMillan N. A. J., & Idris A. (2022). Hyperactivating p53 in Human Papillomavirus-Driven Cancers: A Potential Therapeutic Intervention. Molecular Diagnosis & Therapy, 26(3), 301–308. doi: 10.1007/s40291-022-00583-5 35380358 PMC9098605

[7] Habbous S., Pang V., Eng L., Xu W., Kurtz G., Liu F. F., et al. (2012). p53 Arg72Pro polymorphism, HPV status and initiation, progression, and development of cervical cancer: A systematic review and meta-analysis. Clinical Cancer Research, 18(23), 6407–6415. doi: 10.1158/1078-0432.CCR-12-1983 23065429

[8] Qiao F., Tu M., & Liu H. (2021). Role of MALAT1 in gynecological cancers: Pathologic and therapeutic aspects (Review). Oncology Letters, 21, 333. doi: 10.3892/ol.2021.12594 33692865 PMC7933745

[9] Lin T., Hou P.F., Meng S., Chen F., Jiang T., Li M.L., et al. (2019). Emerging roles of p53 related lncRNAs in cancer progression: A systematic review. International Journal of Biological Sciences, 15(6), 1287–1298. doi: 10.7150/ijbs.33218 31223287 PMC6567798

[10] Kldiashvili E., Iordanishvili S., & Metreveli T. (2021). High SOX2 expression as an indicator of the potential involvement of cancer stem cells in the development of cervical cancer among females with HPV infection. CellR4, 9, e3251. doi: 10.32113/cellr4_20219_3251

[11] Agreda PM, Beitman GH, Gutierrez EC, et al. (2013). Long-term stability of human genomic and human papillomavirus DNA stored in BD SurePath and Hologic PreservCyt liquid-based cytology media. Journal of Clinical Microbiology, 51(8), 2702–2706. doi: 10.1128/JCM.00759-13 23678069 PMC3719621

[12] Zavridou M.; Mastoraki S.; Strati A.; Tzanikou E.; Chimonidou M.; Lianidou E. (2018). Evaluation of preanalytical conditions and implementation of quality control steps for reliable gene expression and DNA methylation analyses in liquid biopsies. Clinical Chemistry, 64(10), 1522–1533. doi: 10.1373/clinchem.2018.292318 30018056

[13] Ji P, Diederichs S, Wang W, Boing S, Metzger R, Schneider PM. et al. (2003). MALAT-1, a novel noncoding RNA, and thymosin beta4 predict metastasis and survival in early-stage non-small cell lung cancer. Oncogene, 22(39), 8031–8041. doi: 10.1038/sj.onc.1206928 12970751

[14] Yamada K, Kano J, Tsunoda H, Yoshikawa H, Okubo C, Ishiyama T. et al. (2006). Phenotypic characterization of endometrial stromal sarcoma of the uterus. Cancer Science, 97(2), 106–112. doi: 10.1111/j.1349-7006.2006.00147.x 16441420 PMC11158577

[15] Guffanti A, Iacono M, Pelucchi P, Kim N, Solda G, Croft LJ. et al. (2009). A transcriptional sketch of a primary human breast cancer by 454 deep sequencing. BMC Genomics, 10, 163. doi: 10.1186/1471-2164-10-163 19379481 PMC2678161

[16] Guo F., Li Y., Liu Y., Wang J., Li Y., & Li G. (2010). Inhibition of metastasis-associated lung adenocarcinoma transcript 1 in CaSki human cervical cancer cells suppresses cell proliferation and invasion. Acta Biochimica et Biophysica Sinica, 42(4), 224–229. doi: 10.1093/abbs/gmq008 20213048

[17] Yang L, Lin C, Liu W, Zhang J, Ohgi KA, Grinstein JD. et al. (2011). ncRNA-and Pc2 methylation-dependent gene relocation between nuclear structures mediates gene activation programs. Cell, 147(4), 773–788. doi: 10.1016/j.cell.2011.08.054 22078878 PMC3297197

[18] Tripathi V, Shen Z, Chakraborty A, Giri S, Freier SM, Wu X. et al. (2013). Long noncoding RNA MALAT1 controls cell cycle progression by regulating the expression of oncogenic transcription factor B-MYB. PLoS Genetics, 9(7), e1003368. doi: 10.1371/journal.pgen.1003368 23555285 PMC3605280

